# Reply to Foster, T.P.; Schatz, D. Comment on “Lombardo et al. The Impact of Insulin-Induced Lipodystrophy on Glycemic Variability in Pediatric Patients with Type 1 Diabetes. *Children* 2022, *9*, 1087”

**DOI:** 10.3390/children12020112

**Published:** 2025-01-21

**Authors:** Fortunato Lombardo, Bruno Bombaci, Angela Alibrandi, Giulia Visalli, Giuseppina Salzano, Stefano Passanisi

**Affiliations:** 1 Department of Human Pathology in Adult and Developmental Age “Gaetano Barresi”, University of Messina, Via Consolare Valeria 1, 98124 Messina, Italy; fortunato.lombardo@unime.it (F.L.); brunobombaci@gmail.com (B.B.); giulia.vsl@hotmail.com (G.V.); gsalzano@unime.it (G.S.); 2 Department of Economics, Unit of Statistical and Mathematical Sciences, University of Messina, 98124 Messina, Italy; aalibrandi@unime.it

We appreciate the insightful comment by Foster and Schatz [1] regarding our research on insulin-induced lipodystrophies published in 2022 [2].

As highlighted in the comment, greater focus on clinical examination during outpatient visits is crucial for detecting cutaneous complications related to insulin therapy, as is providing appropriate training on insulin administration site rotation, regardless of the treatment modality.

We wish to share our concerns regarding lipoatrophy. Although its prevalence has drastically decreased since the introduction of human purified insulin, this skin complication still represents a serious issue for some individuals with diabetes [3,4,5]. In our clinical practice, emerging cases have recently been observed (Figure 1). Despite several pathogenetic theories, including the autoimmune hypothesis [6], the pathogenesis of lipoatrophy remains unclear. This complication can compromise the efficacy of insulin therapy and, given the absence of specific treatment strategies, may be challenging to reverse [7].

We believe further studies also investigating the histopathological characteristics of lipoatrophy are essential to clarify the pathogenetic basis of this condition and to develop novel, effective, preventive, and therapeutic strategies.

## Figures and Tables

**Figure 1:**
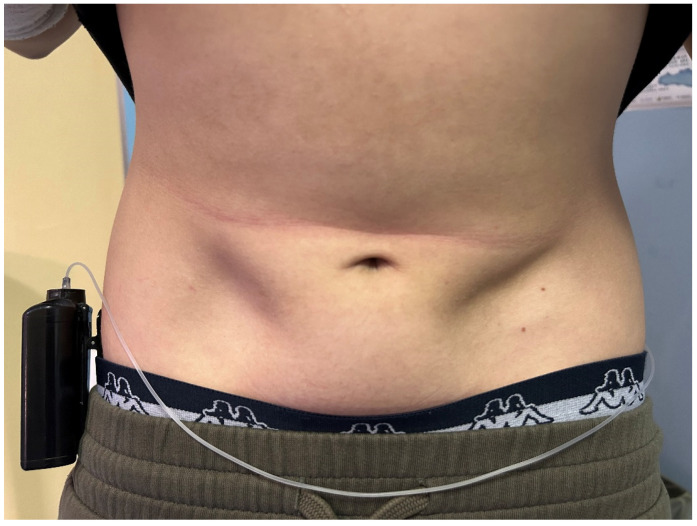
Child with severe lipoatrophy at the site of a previous infusion set insertion for an insulin pump.
